# The Burden and Prevention of Human Papillomavirus (HPV) Infections and Cervical Cancer in India: A Literature Review

**DOI:** 10.7759/cureus.72435

**Published:** 2024-10-26

**Authors:** Vinit N Deshmukh, Satish Patil, Dilip D Hinge

**Affiliations:** 1 Department of Molecular Biology and Genetics, Krishna Vishwa Vidyapeeth (Deemed to be University), Karad, IND; 2 Department of Microbiology, Krishna Institute of Medical Sciences, Karad, IND

**Keywords:** cancer vaccine, disease burden, human papillomavirus (hpv), prevalence, uterine cervical cancer

## Abstract

Human papillomavirus (HPV) is a widespread viral infection affecting the reproductive tract and is associated with multiple types of cancer. It is a significant global health concern, with cervical cancer being one of the most common cancers affecting women worldwide. HPV infection has been found in both married and unmarried women. The burden of cervical cancer is particularly high in middle- and low-income countries, where HPV vaccination and screening programs are often limited.

India faces a substantial challenge with cervical cancer and HPV infection. Cervical cancer ranks as one of the leading cancers among women in India. Studies over recent decades have indicated varying levels of HPV prevalence in the general female population in India.

Recognizing the importance of addressing this issue, the Government of India has prioritized cervical cancer elimination as a national public health goal. A strategic plan has been launched to increase cervical cancer screening coverage in adult women and HPV vaccination coverage in girls.

This review examines literature published from 2000 to 2023 on the epidemiology of cervical cancer and HPV in India. It also explores the development of prevention strategies, focusing on cervical screening and HPV vaccination programs. Government policy documents were analyzed to understand the national strategic vision and targets. The review concludes by discussing ongoing challenges and future directions for cervical cancer elimination efforts in India.

## Introduction and background

Human papillomavirus (HPV) infection encompasses a group of over 200 known genotypes of HPV virus types that can infect human skin and mucous membranes [1]. While HPV infections are common and often cleared by the body's immune system, certain high-risk types can persist potentially leading to cervical cancer, the second most prevalent cancer in Indian women [2,3]. In 2018, the World Health Organization (WHO) reported an estimated 96,922 new cases and 60,078 deaths because of cervical cancer in India, representing 16.5% of the global burden [4]. HPV, a sexually transmitted infection, belongs to the *Papillomaviridae* family. It is a small, non-enveloped, circular double-stranded DNA virus with a 52-55 nm diameter and approximately 8000 base pairs encased in a protein capsid composed of 72 capsomers [5,6]. Alpha papillomaviruses are responsible for about 5% of cancer occurrences worldwide [7]. Despite the established causal relationship between cervical cancer and HPV demonstrated in numerous global studies, there is limited knowledge about the prevalence of specific HPV types and their persistence patterns in the Western Maharashtra region of India [8]. HPV genotypes are known to vary by population and geographic area, underscoring the importance of local epidemiological data [9]. Cervical cancer, while being the most important common cancer among Indian women, is both curable and preventable if detected early and managed effectively [10,11]. High-risk HPV detection has the potential to serve as a valuable tool in identifying women at risk of developing cervical cancer [12]. It is an urgent need to determine the prevalence of both asymptomatic and symptomatic cervical HPV infections in the local population [13]. This review synthesizes the evidence from the literature published from 2000 to 2023 on the epidemiology of cervical cancer and HPV in India. It also reviews the evolution of prevention strategies, specifically cervical screening and HPV vaccination programs. The Government of India reviewed policy documents to assess the national strategic vision and targets. The review concludes with a discussion of remaining barriers and future directions to achieve the target of eliminating cervical cancer in India.

## Review

Methods

A search for literature was conducted in December 2023 to identify the relevant studies on cervical cancer and HPV in India published from 2000 to 2023. PubMed, Google Scholar, UniProt, HPV Database, and manual review were searched of reference lists using the following terms: "HPV", "human papillomavirus", "cervical cancer", "India", "epidemiology", "prevalence", "vaccination", and "screening". Relevant peer-reviewed articles, population-based surveys, statistics, and policy documents from reputable and different health organizations were included. Abstracts were reviewed for relevance, and full-text articles were analyzed to synthesize data on HPV prevalence, genotype distribution, HPV vaccination coverage, cervical cancer screening, costs and cost-effectiveness, policy targets, and challenges for HPV prevention in India. 

Research question

What is the current state of HPV and cervical cancer in India, including prevalence, genotype distribution, vaccination coverage, screening practices, and prevention challenges?

Inclusion criteria

Inclusion criteria were as follows: relevant peer-reviewed articles, population-based surveys, statistics from reputable health organizations, policy documents from reputable health organizations, and studies published from 2000 to 2023 and focusing on cervical cancer and HPV in India.

Exclusion criteria

Studies not specific to India, publications before 2000 and after 2023, non-peer-reviewed articles or unreliable sources, and studies focusing solely on other HPV-related cancers (e.g., oral, cervical) were excluded.

**Figure 1:**
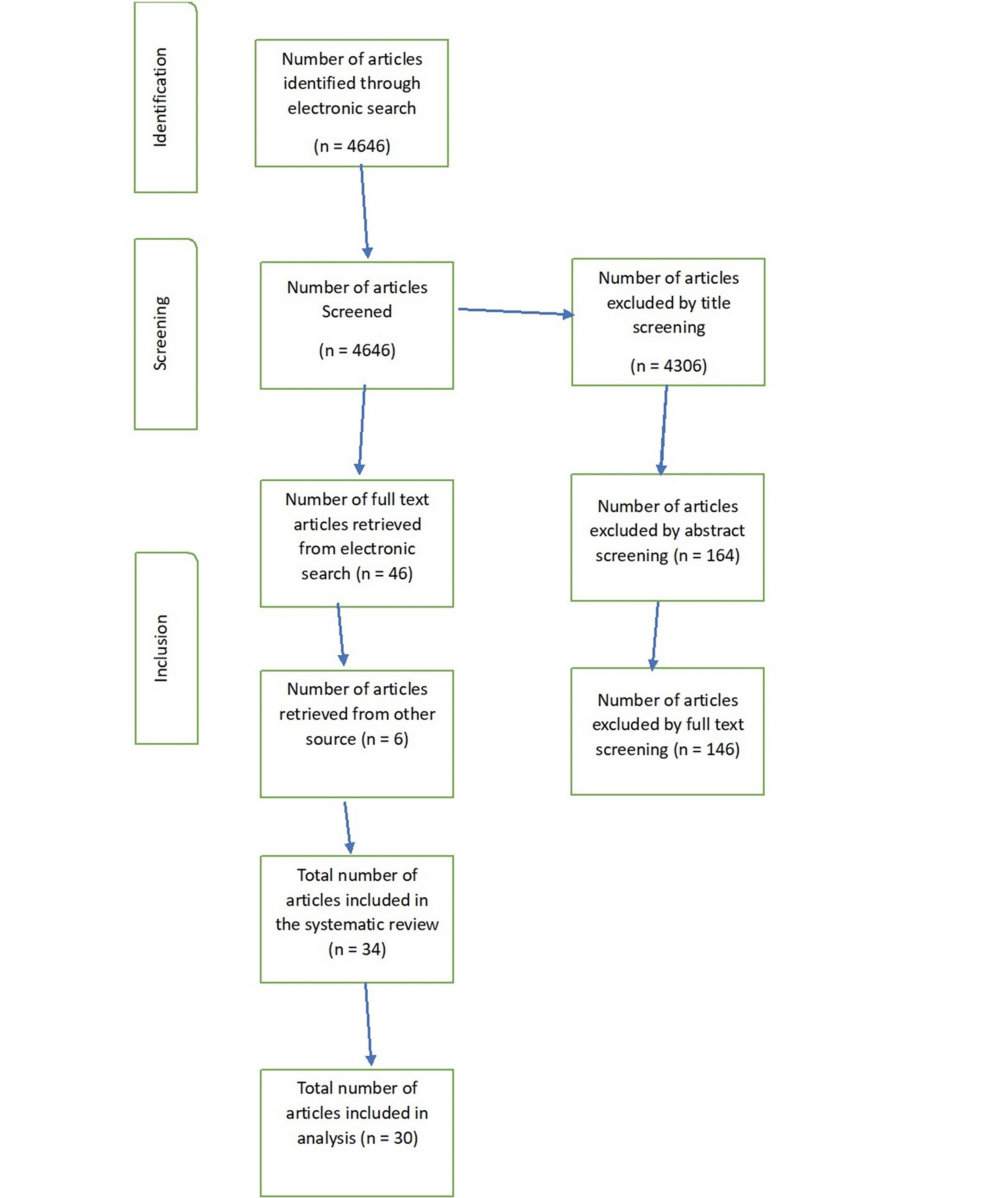
Flowchart

Figure 1 shows the flowchart of the study.

Epidemiology of HPV infections: prevalence 

The genotype distribution and prevalence of HPV in India vary widely depending on the region, type of the sample, and method of detection [1]. A meta-analysis of 3690 women from different states reported an overall HPV infection rate of 29.97%, with HPV 6, 11, 16, 58, and 18 being the common genotypes [2,3]. HPV 6 and 11 are low-risk types that cause genital warts, while HPV 18 and 16 are high-risk types, and HPV 18 and 16 types are responsible for nearly about 70% of cases of cervical cancer in women globally [4,6]. HPV 58 is another high-risk type that is more prevalent in Asia than in other regions [5-7]. Another study of 595 women without and with cervical lesions found a higher HPV prevalence of 60.33%, with HPV 18, 16, and 51 being the most prevalent [8,9]. The study also found that HPV 18 and 16 were remarkably associated with invasive cervical cancer (ICC) cases, while HPV 51 was more common in inflammatory smears [10,11]. HPV 51 high-risk type is a probable that has been detected in precancerous lesions and cervical cancer [12-14]. A longitudinal study of 497 women from rural Maharashtra observed an HPV prevalence of 36.4% over six years, with HPV 16 and 31 being the most frequent [13]. The study also measured the acquisition, persistence, and clearance of HPV infection over time. The new HPV acquisition rate was 5.6 per 1000 person-months of observation (PMO), the highest for HPV genotype 16 (1.1 per 1000 PMO). The type-specific clearance rates ranged between 2.9 and 5.5 per 100 PMO, with HPV 35 (62.5%) and 52 (25%) showing the highest persistence [14]. HPV 35 and 52 are also high-risk genotypes that have been linked to cervical cancer [1,15]. Recently, one START-UP study conducted in 2019-2020 in the city among 899 women aged 16-26 years across seven sites found that the overall HPV prevalence was 13.3% [16]. Another recent multicentric study conducted in the year 2017 showed the HPV infection prevalence was 10.3% among 3,542 women aged 17-57 years across 10 sites [17]. In conclusion, the population-based studies estimate the prevalence of HPV infection among general women in the population in India to range from 7.5% to 16.9% ​[18]. The mean age of participants in these studies ranged from 35 to 39 years. HPV infection prevalence tended to be higher in city (urban) areas and among higher socioeconomic groups [11,12,14,19] (Table 1).

**Table 1:** Prevalence of HPV infection in India HPV: human papillomavirus

Region	Prevalence of HPV infection in India (%)	Age group
Urban	13.3	16-26
Rural	10.3	17-57
Maharashtra	36.4	16-49

High-risk HPV types

**Figure 2:**
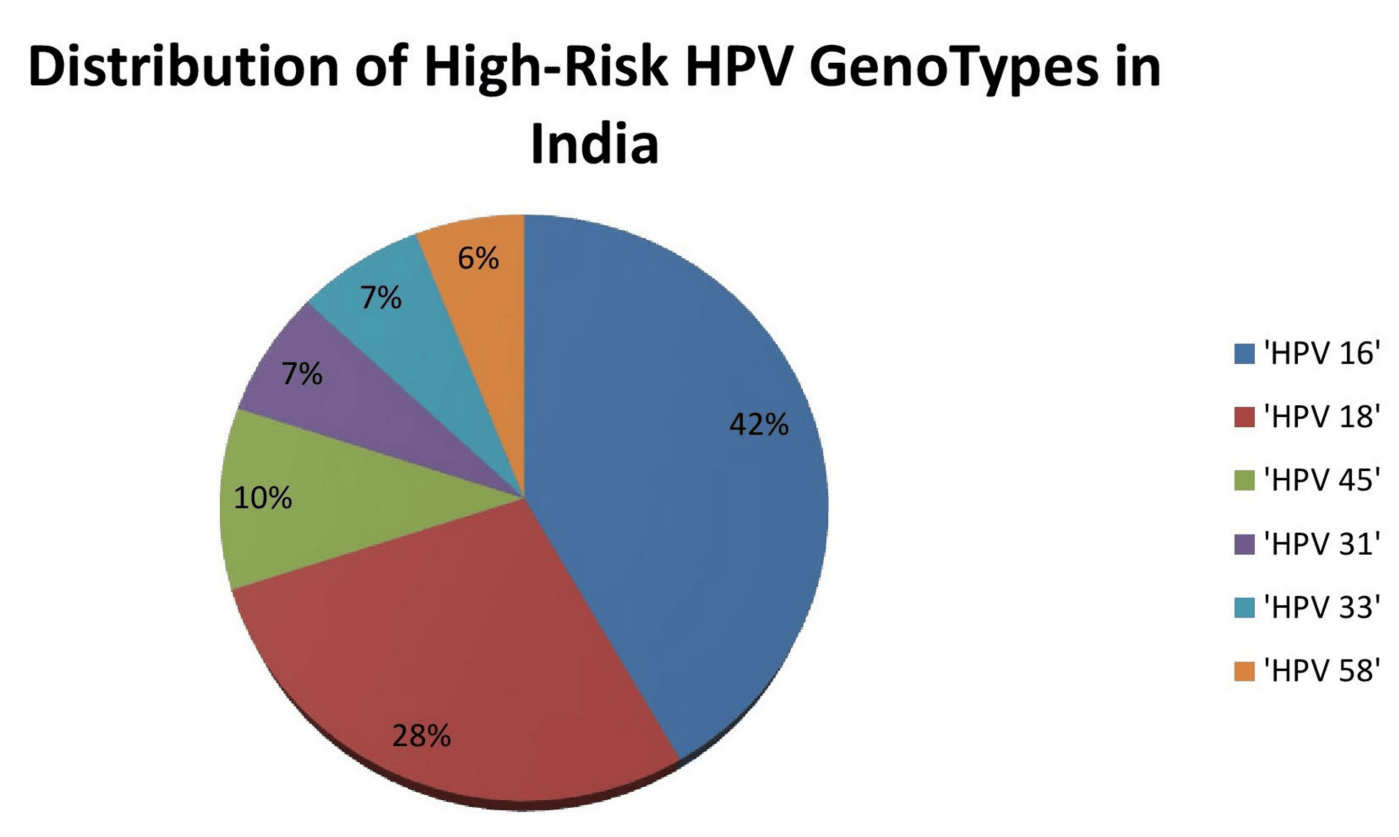
Distribution of high-risk HPV genotypes in India HPV: human papillomavirus

Among HPV-positive women, high-risk types 18 and 16 consistently account for approximately 70% of infections in population-based studies [18,19]. Other frequent high-risk types include 45, 31, 33, and 58. HPV 16 remains the important common genotype, contributing to 41.7% of infections in the START-UP study [20]. HPV 18 and 16 are responsible for the greatest cancer risk and contribute to around 70% of cervical cancers in India ​[21] (Figure 2).

Cervical cancer burden 

**Figure 3:**
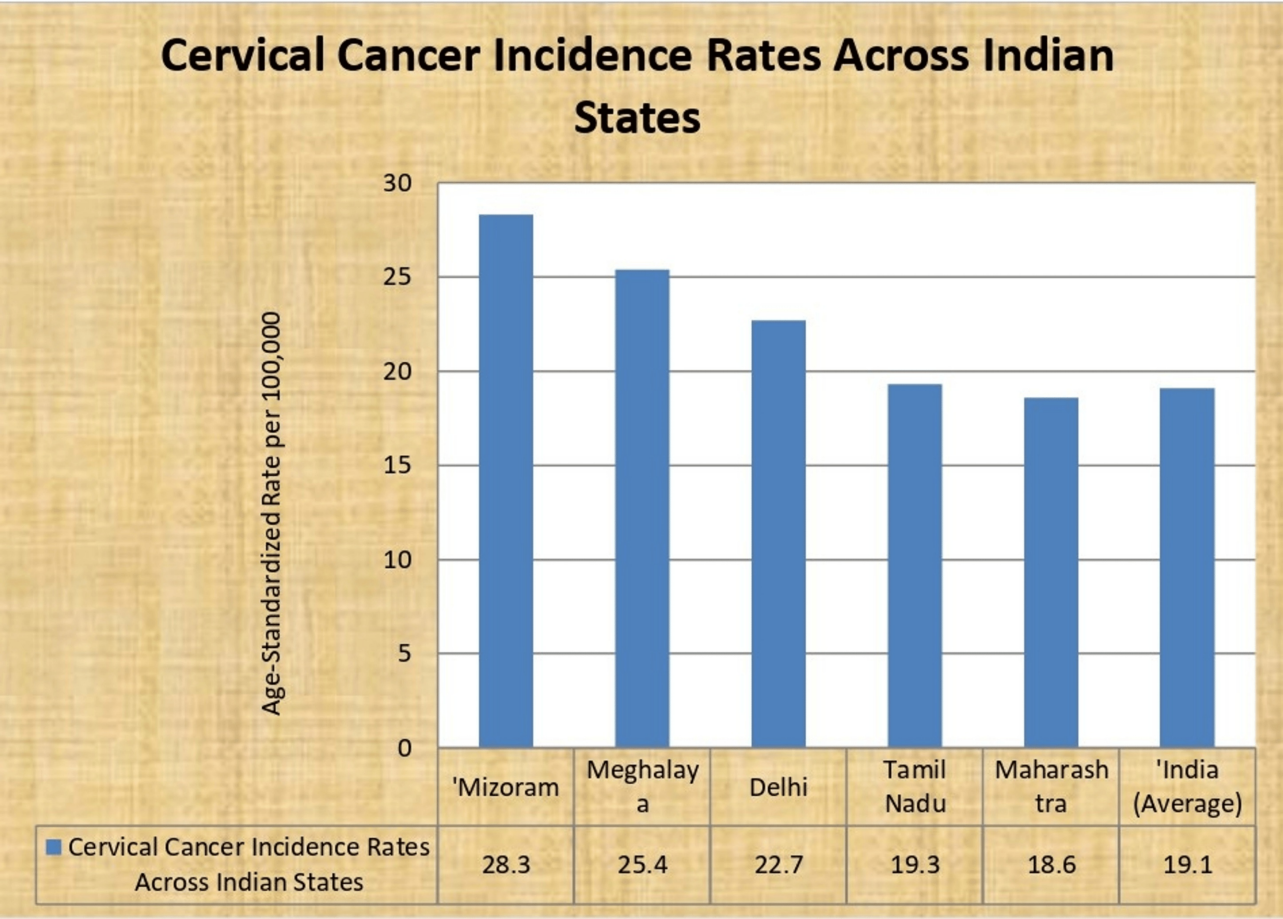
Cervical cancer incidence rates across Indian states

Cervical cancer is the second most common cancer in women in India after breast cancer. India accounted for nearly one-fifth (18.6%) of the global cervical cancer mortality burden in 2018 ​[19,21]. An estimated 96,000 new cases and 60,000 cervical cancer deaths occurred in India in 2018 ​[21]. The incidence rates of cervical cancer are highest in the states of Mizoram, Meghalaya, and Delhi [22]. Age-standardized incidence rates have shown a decline in successive National Cancer Registry Programme reports, from 22.9 per 100,000 women during 2008-2011 to 19.1 per 100,000 women during 2012-2014 [23]. However, cervical cancer still disproportionately impacts women in India during their reproductive years compared to high-income regions. The peak age of diagnosis is 49-65 years, with 10% of cases diagnosed under age 35 years [22,23] (Figure 3).

Prevention strategies for HPV and cervical cancer in India 

HPV Vaccination 

HPV vaccination was first recommended for teenage girls aged 10-12 years in India in 2008 ​[24]. Both quadrivalent and bivalent vaccines have been available. By 2017, the national advisory body recommended the inclusion of HPV vaccination in the Universal Immunization Program which provides all recommended childhood vaccines free of cost across India ​[25]. However, HPV vaccination has not yet been integrated, and India's national HPV vaccine coverage remains very low. Multiple small studies have found HPV vaccine coverage to be less than 2% among young girls across India including states like Haryana, Punjab, Tamil Nadu, and Maharashtra ​[17,25]. The Fourth National Family Health Survey (2019-2020) found only 1.3% of girls aged 15-18 years had received the full dose of the HPV vaccine ​[25]. Sub-national variation exists with higher self-reported coverage of 2.7% in urban areas compared to 1.1% in rural areas. Key barriers to HPV vaccination in India include the high cost of the vaccine, low awareness about cervical cancer and HPV among the public, lack of physician recommendation, infrastructural challenges for vaccine delivery, and vaccine hesitancy. 

Screening of Cervical Cancer

Screening of cervical cancer programs in India has primarily relied on opportunistic screening using either visual inspection with acetic acid (VIA) or pap smear. Historically, national screening guidelines recommended a single pap test between ages 30 and 65 years at a 10-year interval. In 2020, the same national screening guidelines changed again the recommended age range for screening which was revised to 21-65 years with a preference for HPV DNA testing as the primary screening modality ​[26,27]​. However, screening coverage remains very low. The Fourth National Family Health Survey in 2019-2020 found that only 13.2% of women aged 15-49 years had ever undergone screening for cervical cancer ​[28,29]. Barriers to improving screening uptake include limited awareness, lack of women's agency, inadequate health infrastructure, shortage of trained providers, and sociocultural barriers ​[29].

Plan of National Strategic for Cervical Cancer Elimination

In 2020, the Government of India launched a plan of National Strategic for Cervical Cancer Elimination aligned with the WHO's global strategy ​[1-4,16,28]. Key targets for 2030 include 90% HPV vaccination coverage for girls aged 9-14 years and 70% screening coverage targeted for women aged 30-65 years. The recommended technologies are HPV DNA testing for primary screening and thermal ablation for the treatment of precancerous lesions. The strategic plan emphasizes partnerships between the Government of India and the private sector to complement public health services. It highlights the need for intense social and behavioral change communication to spread awareness and address vaccine hesitancy. Continued political commitment, decentralized planning, adequate financing, and robust monitoring will be crucial to translate these targets into impact.

Discussion

In past decades, India has made gains in implementing organized cervical cancer control programs. However, the country still faces substantial barriers to improving HPV vaccine uptake and increasing screening coverage. Achieving the WHO call for action to eliminate cervical cancer by 2030 will require concerted efforts to scale up vaccination, expand screening and diagnostic services, strengthen recording and surveillance systems, increase public awareness, and foster innovative delivery partnerships. India has a critical window of opportunity with its demographic dividend of the young population. Investing in life-saving HPV vaccines and cervical cancer prevention strategies will protect the lives of generations of women to come.

## Conclusions

These studies highlight the need for effective HPV screening and vaccination programs in India, especially in rural areas where access to healthcare is limited. HPV screening can detect HPV infection and cervical abnormalities before they progress to cancer and allow for timely treatment. HPV vaccination can prevent infection by the most common and high-risk types and reduce the mortality and incidence of cervical cancer. The WHO recommends HPV infection vaccination for girls aged 9-14 years before they become sexually active. India has not yet introduced HPV vaccination in its national immunization program, but some states have implemented pilot projects or campaigns to vaccinate adolescent girls. Increasing the awareness and acceptance of HPV vaccination and screening among the general public and healthcare providers is crucial for controlling and preventing cervical cancer in India.
